# Cataract Development by Exposure to Ultraviolet and Blue Visible Light in Porcine Lenses

**DOI:** 10.3390/medicina57060535

**Published:** 2021-05-27

**Authors:** Robin Haag, Nicole Sieber, Martin Heßling

**Affiliations:** Institute of Medical Engineering and Mechatronics, Ulm University of Applied Sciences, 89081 Ulm, Germany; Nicole.Sieber@thu.de

**Keywords:** cataract, blue light, UV radiation, porcine lens, UVA, UVB

## Abstract

*Background and Objectives*: Cataract is still the leading cause of blindness. Its development is well researched for UV radiation. Modern light sources like LEDs and displays tend to emit blue light. The effect of blue light on the retina is called blue light hazard and is studied extensively. However, its impact on the lens is not investigated so far. *Aim*: Investigation of the impact of the blue visible light in porcine lens compared to UVA and UVB radiation. *Materials and Methods*: In this ex-vivo experiment, porcine lenses are irradiated with a dosage of 6 kJ/cm^2^ at wavelengths of 311 nm (UVB), 370 nm (UVA), and 460 nm (blue light). Lens transmission measurements before and after irradiation give insight into the impact of the radiation. Furthermore, dark field images are taken from every lens before and after irradiation. Cataract development is illustrated by histogram linearization as well as faults coloring of recorded dark field images. By segmenting the lens in the background’s original image, the lens condition before and after irradiation could be compared. *Results*: All lenses irradiated with a 6 kJ/cm^2^ reveal cataract development for radiation with 311 nm, 370 nm, and 460 nm. Both evaluations reveal that the 460 nm irradiation causes the most cataract. *Conclusion*: All investigated irradiation sources cause cataracts in porcine lenses—even blue visible light.

## 1. Introduction

Worldwide context. Cataract describes the opacification—the loss of transparency—of the eye lens [1]. It is well documented that cataract development is a worldwide circumstance and the main cause of moderate to severe vision impairment (MSVI) and even blindness [2,3,4] and is therefore of tremendous significance [5].

In 1990, with a global population of 5.8 billion people [6], about 31.8 million people were blind, and 172 million people had an MSVI [4] compared to forecast data for 2020 when 38.5 million people [3] up to 43.2 million people [2] were estimated to be blind and 237.08 [4] to 295.3 [2] million people were estimated to suffer from an MSVI. The above-documented statistics represent symptoms (blindness, MSVI) that can be subdivided into different illnesses such as cataracts, glaucoma, uncorrected refractive error, and more. This division leads to a rate of 36.67% of blind people being blind by cataract in 1990 [3]. The division in 2020 is 34.73% of the blind people being blind by cataract, which is still the leading cause of blindness [3].

Cataract types. A cataract is clinically distinguishable by its color, morphology, and location. The three main types are nuclear, cortical, and posterior subcapsular cataracts [1]. A nuclear cataract (NUC) starts in the center (nucleus) of the lens spreading outwards over the cortex to the equator until the whole lens is opaque. The cortical cataract (COR) begins at the cortex, growing towards the nucleus in a spiky way. In a posterior subcapsular cataract (PSC), opacification occurs on the posterior side of the lens near its capsule [1,7,8]. Another common cataract is the senile or age-related cataract, which mostly becomes visible at the age of 70 or older [7,9].

Risk factors. The known risk factors are dividable in acquired factors and congenital factors [7,10]. Some of the acquired factors are smoking and alcohol consumption, exposure to radiation (especially UV radiation), vitamin and protein deficiency, high blood pressure, increasing age, systemic diseases (such as diabetes mellitus), and other ocular diseases [1,9,10,11]. These factors are mostly influenceable by personal caretaking. The congenital factors are already present at birth or develop due to genetic predisposition. A common gene mutation-based cataract is a Y-shaped sutural cataract [12,13].

LED, other light sources, and blue light hazards. Since the mid-1990s, there is a profound change in available light sources. Beginning with the first white light-emitting diodes (LEDs), the world switched from conventional illumination to generally widespread LED [14]. Today LEDs are everywhere, such as displays, spotlights, street lightning, room lightning, cars, and optical signals for telecom fibers [15,16]. In 2025, 90% of all lightbulbs will be LEDs, compared to 1% in 2010 [17]. Due to the specific spectrum of especially blue and white LEDs with a high peak in the blue wavelength range from 400 nm to 490 nm, the radiation has a phototoxic effect on the retina. It also applies to other light sources, which emit light in this spectral range too. This association between the blue light spectrum and the phototoxic effect on the retina is called *blue light hazard* [18]. Intense irradiation with blue light leads to retinal tissue inflammation and retinal damage [17,19]. The impact of the blue light hazard is not limited to LEDs. Fluorescent tubes and energy-saving lamps also have a significant blue component and emit in the violet and ultraviolet spectral range [20], which is harmful to the retina. While the blue light hazard is largely investigated for its effect on the retina, studies about the lens are missing.

Radiation and lens properties. Mechanisms that damage the eye tissue are similar for retina and lens [21,22]. Therefore, blue light and radiation in general, which harms the retina, might also cause damage to the lens. Kamari et al. [23] suggest a categorization by mechanisms and their pathways causing lens opacification consisting of different impacts: oxidative stress, phototoxicity, crystalline proteins, tryptophan, and apoptosis [23]. According to the Grotthus–Draper law, radiation influences biochemical mechanisms when absorbed by the material [24]. Therefore, the absorption spectrum of the biological material and the wavelength of the radiation gives insight into the above-mentioned possible damaging pathways. Radiation in the wavelength range between 100 and 780 nm is categorized in different ranges as UVC (100–280 nm), UVB (280–315 nm), UVA (315–400 nm), and visible light (380–80 nm), including the blue light hazard which covers the range of 400–490 nm [18]. The ozone layer naturally absorbs radiation from the UVC spectrum emitted by the sun, or if artificially present due to low-pressure mercury vapor lamps or other sources, the cornea completely absorbs it. Hence, the harmful radiation for the lens eye tissue begins with the UVB spectrum. UVB radiation at 300 nm impacting the eye is absorbed up to 92% by the cornea, followed by 6% absorption in the aqueous humor and 2% absorption in the lens [25].

Moreover, 37% of UVA radiation at 340 nm is absorbed by the cornea, 14% by the aqueous humor, and 48% by the lens [25]. In general, lens transparency is heavily age-dependent. Newborns have a very high overall transparency, notably in the UVA and UVB spectrum, whereas transparency decreases [26]. The two optical effects causing vision impairment resulting from cataract development are absorption and light scattering of visible light in the eye lens [27].

Studies and Experiments. Most research regarding in vivo experiments is performed with rodents, especially rats [19,24,28,29,30,31,32,33]. The irradiation dosage needed to induce cataracts in rat eyes with a wavelength of 300 nm is determined to be 2.2 kJ/m^2^ [32]. Other animals used for cataract research, but less frequently, are pigs and cows. Most experiments on porcine and bovine lenses are performed ex-vivo [27,34,35,36,37,38,39,40,41]. Common methods applied for evaluation are slit lamps (photography), fluorescence measurements, and chromatographic procedures.

Dark field images. Dark field microscopy generates images with a black background and an object’s visible structure resulting from light scattering. This method can monitor cataracts in lenses in an in-vitro setup. The resulting dark field images can be further analyzed. One aspect is the kind of cataract that developed. Other aspects are the amount of light scattering and the size of the cataract, which is calculable to a transmission value of the whole lens.

The trial/experiment. This experiment gives a first impression of blue light (460 nm) affecting the porcine lens ex-vivo. For comparison, further porcine lenses are irradiated with known cataractogenesis wavelengths like 311 nm (UVB) and 370 nm (UVA). To evaluate the effect of this radiation on the porcine lens, transmission spectra are recorded. Additionally, a new dark field imaging technique documents the opacification of the lenses.

## 2. Materials and Methods

Experimental Setup. Ex-vivo porcine eyes were obtained from the local abattoir and originate from 6-month-old pigs. The experiment started on the same day of slaughter and enucleation of the porcine eye. During the short transport from the abattoir to the laboratory, the eyes were kept in a balanced salt solution (BSS) for a similar physiological surrounding.

**Figure 1:**
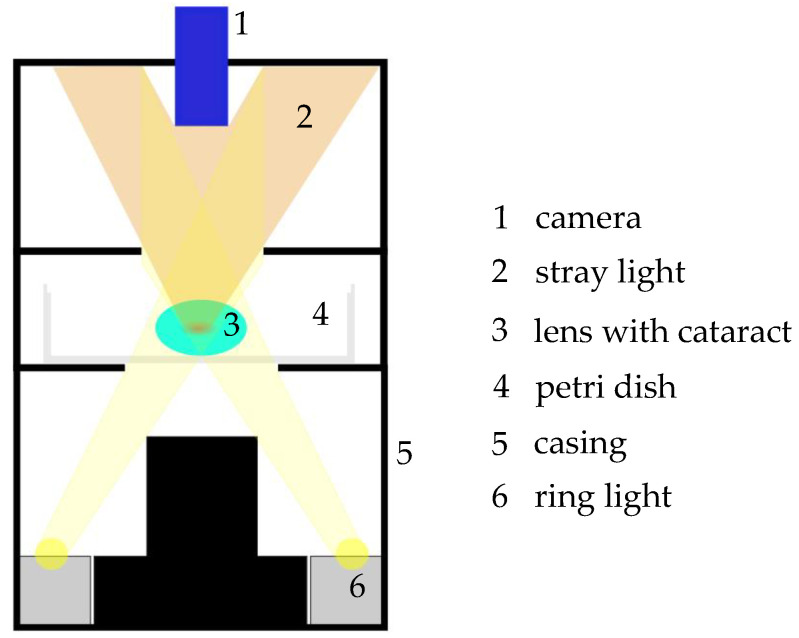
Schematic drawing of the custom-made dark field image setup. A ring light (6) illuminates the sample from below. The light that passes the sample in a straight line is not detected by the camera (1), whereas scattered light (2) from insight the sample is detected by the camera from above. With this setup, cataractogenic structures can be made visible.

In the laboratory, the porcine lenses were carefully extracted with chirurgical instruments and stored in BSS. Lenses with obvious damage were sorted out. The transparency of the lenses was measured from 380 nm to 780 nm with the microtiter plate reader BMG Labtech CLARIOstar (Allmendgrün, Germany) in a 6 x 8-microtiter plate COSTAR48 (Corning, NY, USA). During these measurements, the lenses were kept in BSS too. As reference measurement, pure BSS was applied. The lenses were carefully lifted out of the microtiter plate and placed in a petri dish with BSS. Afterward, a dark field image was taken from the lenses with a custom-made setup presented in Figure 1. The applied camera was an IMAGINGSOURCE DMK 33UX265 (Bremen, Germany), and the image format was 1920 x 1080 pixels as a Windows Bitmap (.bmp) grey-image. All images were taken with the same settings.

**Figure 2:**
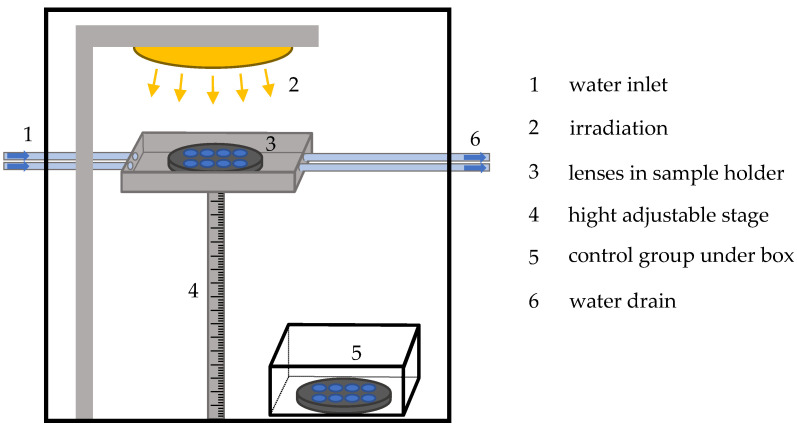
Schematic drawing of the experimental setup. A closed box prohibits radiation reaching the outside of the experimental box. To prevent overheating of the lenses cold water circles ((1) and (6)) around the sample holder (3). The radiation source (2) irradiates the sample from above. With the height-adjustable stage (4), the distance between lenses and radiation source is regulated. The control group (5) is located under a box shielded from irradiation.

For irradiation, the lenses were placed in a specially formed lens sample holder (see Figure 2). The different radiation sources were a PL-S 9W UV-A/2P 1CT/6X10CC lamp for the UVA radiation at 370 nm and a PL-S 9W/01/2P 1CT/6X10BOX lamp for the UVB radiation at 311 nm, which were both from Philips (Amsterdam, The Netherlands). To narrow the emitting spectrum and block visible light from UV lamps, the filter “HU01” from HEBO (Aalen, Germany) was applied. For irradiation with 460 nm, two XLamp XQ-E High-Intensity Royal Blue LEDs from Cree (Durham, NC, USA) were used to obtain a more homogenous irradiation distribution. These LEDs were connected in a parallel circuit and operated with a power supply unit at an electrical current of 2 A. All lenses were irradiated with a dosage of 6 kJ/cm^2^. This high dosage was chosen according to other experiments inducing cataracts with UVB (about 300 nm) radiation with a dosage of 0.8 J/cm^2^ [29]. The biochemical impact of blue light is about four orders of magnitude smaller than UVB radiation’s impact [42]. Therefore, 6 kJ/cm^2^ was applied. All lenses were irradiated for 24 h. During irradiation, the lenses were kept in a solution mixture of 94% BSS, 4% fetal calf serum, and 2% GVPC (Glycine, Vancomycin, Polymyxin B, Cycloheximide) to reduce the growth of bacteria and other microorganisms.

Furthermore, to inhibit overheating the lenses due to the irradiation, the sample holder was surrounded by cold water (see Figure 2). The water was pumped by a peristaltic pump and was therefore in continuous exchange. A control group of porcine lenses was kept under the same conditions without any irradiation.

After irradiation, the lenses were carefully lifted out of the lens sample holder into a 6 x 8-microtiter plate COSTAR48 (Corning, NY, USA) for transmission measurement. BSS was applied again to prevent the lenses from drying out. Afterward, dark field images were taken as before.

Data. In this investigation a total of 154 lenses were included. Thereof, 24 lenses were irradiated with UVB, 22 lenses were irradiated with UVA, and 28 lenses were irradiated with 460 nm. Meanwhile, 80 lenses served as a control group. Due to obvious damage after irradiation, two lenses each had to be excluded from the UVB and 460 nm irradiated lenses.

The dark field images were evaluated by Matlab R2018b (Natick, MA, USA). The pictures were originally grey images. Therefore, pixels representing the black background had a relatively low value compared to the pixels representing the lens. A Gaussian filter detected the lens in the grey image. Thus, the pixels belonging to the lens were identified, and their values were summed. The resulting value describes a combination of lens properties like transmission and cataract formation. The higher the pixel value, the more cataract development is assumed. A quotient was calculated between the summed pixel value before and after irradiation to describe the lens changes affected by irradiation. For illustrative purposes and highlighting the changes, the dark field images were further edited with histogram linearization and false coloring in Matlab.

## 3. Results

### 3.1. Transmission Measurement

**Figure 3:**
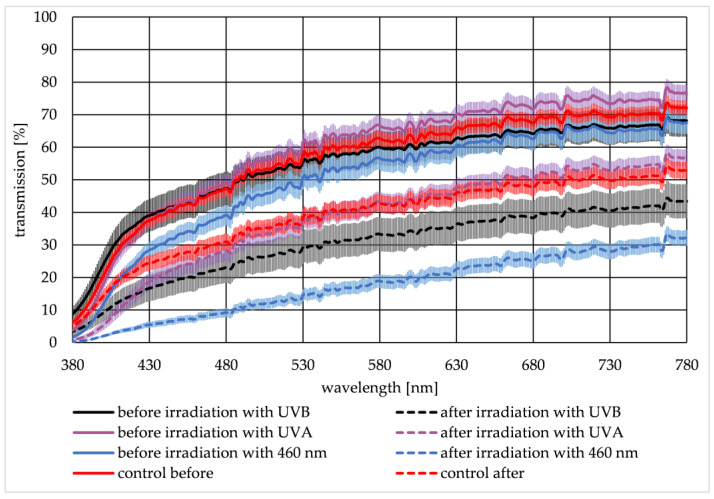
The mean transmission data and the corresponding standard error of the mean (SEM) of porcine lenses are given in the visible spectrum from 380 nm to 780 nm for irradiation with UVB (black curve), UVA (violet curve), 460 nm (blue curve), and the control group (red curve). Before irradiation, measurements are displayed with solid lines, whereas measurements after irradiation are displayed with dashed lines.

Two transmission spectra were recorded for each lens—one spectrum before and the second spectrum after irradiation. Figure 3 illustrates the mean spectrum of the lenses irradiated with UVB (black curve), UVA (violet curve), 460 nm (blue curve), and the control group (red curve), with the corresponding standard error of the mean (SEM). Transmission data before irradiation is given as a solid line, whereas transmission data after irradiation is illustrated with dashed lines. The transmission spectra of the groups before irradiation are very similar. After irradiation, the transmission is lower than the transmission before. For the control group, a decrease in the transmission is also observed. This decrease is an aging effect that generally occurs during the 24 h ex-vivo experiment without any radiation influence. To describe the lenses’ transmission change, a quotient *Q_T_* was calculated (see Equation (1)).

(1) $$Q_{T}=\frac{T\left(\mathrm{after}\right)}{T\left(\mathrm{before}\right)\;}$$

**Figure 4:**
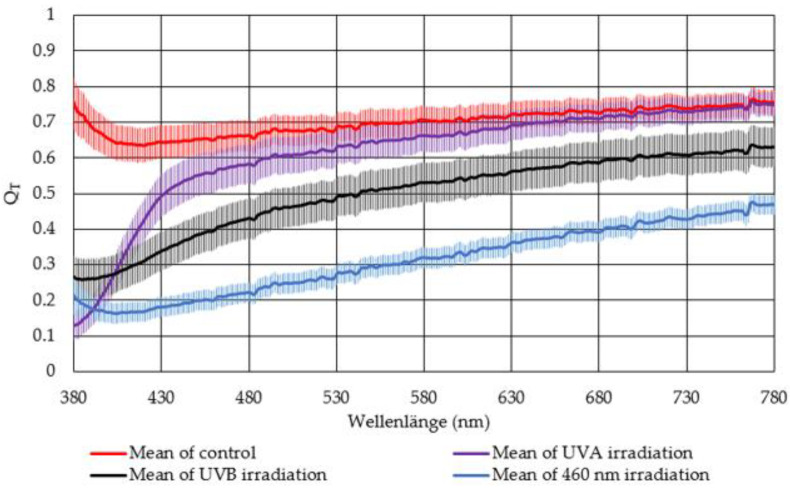
The mean quotient *Q_T_* of the transmission after and before irradiation is illustrated with the standard error of the mean (SEM) for lenses irradiated with UVB (black curve), UVA (violet curve), and 460 nm (blue curve), as well as for the control group (red curve).

Therefore, the transmission value for each wavelength after irradiation *T*(after) was divided by the corresponding transmission value before irradiation *T*(before). *Q_T_* is graphically illustrated in Figure 4. The higher the quotient the less transmission change is observed after irradiation of the lenses. For *Q_T_* = 1, no change in the lens transmission was observed. Therefore, the control group revealed the smallest change in transmission. The smallest change was observed for the irradiated lenses for UVA irradiated lenses, followed by the lenses irradiated with UVB. The largest transmission decrease was observed for lenses irradiated with 460 nm.

### 3.2. Dark Field Images

**Figure 5:**
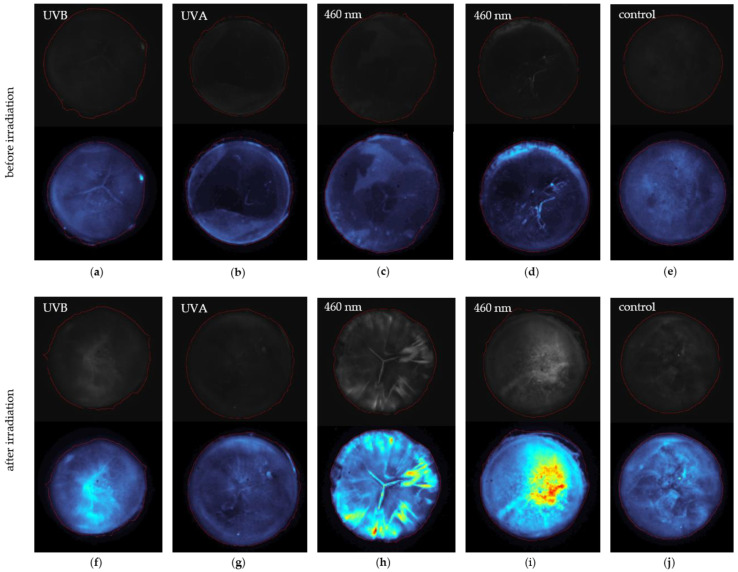
The pictures illustrate some lenses, which developed different kinds of cataracts due to irradiation. In the upper part of each paired picture, the unedited grey images are illustrated. Below there are the false color pictures. All pictures include a circular-shaped red line around the lens, which symbolizes the area Matlab detected as the lens. The upper pictures (**a**–**e**) are taken directly after lens extraction from the eye. The lenses (**f**–**j**) are irradiated. The pictures in (**a**) present a lens before UVB irradiation leading to the pictures in (**f**) after UVB irradiation. An example for a UVA irradiated lens is presented in (**b**,**g**) before and after irradiation, respectively. Lenses before 460 nm irradiation can be seen in (**c**,**d**), whereas (**h**,**i**) represent the irradiated ones. The lens (**e**,**j**) represents the control group.

The original grey images and the edited false color images are presented in Figure 5 upper and lower panels. By comparing the grey images, an effect of the irradiation is noticeable. Before irradiation of lens (a) to (e), the dark field images are dark and do not exhibit any irregularities. After irradiation with 460 nm, the lens (c) developed a COR, and the slightly present Y-cataract gets more visible (h), whereas the lens in (d) developed PSC seen in (i). Images (a) and (f) represent an example of a UVB irradiated lens, and images (b) and (g) illustrate a UVA irradiated lens. A non-irradiated control lens is presented in (e) and (j), with marginal irregularities notable in (j) due to the aging of the lens. The red circle in the images indicates the area, which MATLAB recognizes as the lens. For quantification, the sum of the pixel values within this red circle was calculated. A quotient Q_DFI_ of the sum of the pixels *p* before $\sum_{i=1}^{n}p_{i}\left(\mathrm{before}\right)$ and after irradiation $\sum_{i=1}^{n}p_{i}\left(\mathrm{after}\right)$ is formed for *n* pixels (see Equation (2)).

(2) $$Q_{\mathrm{DFI}}=\frac{\sum_{i=1}^{n}p_{i}\left(\mathrm{before}\right)}{\sum_{i=1}^{n}p_{i}\left(\mathrm{after}\right)\;}$$

**Figure 6:**
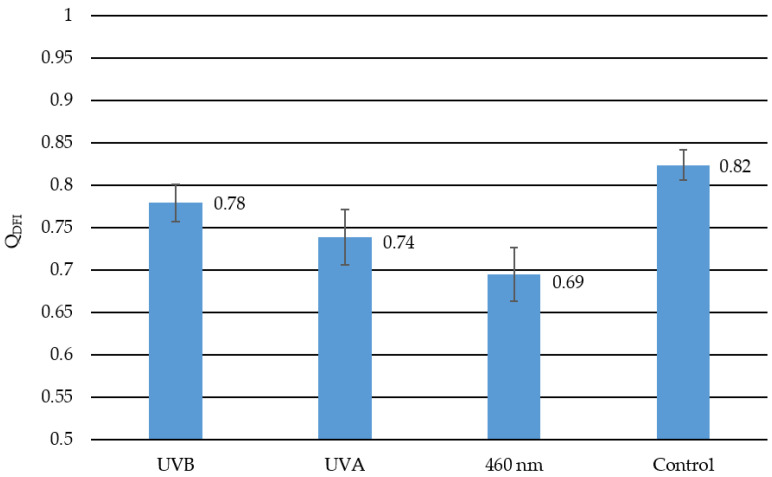
This diagram represents the quotients describing the calculated change from the dark field images for each irradiation and control group. The lower the value the more cataract development was detected. The closer the quotient to one, the less change occurred to the lens. Hence, the control group has the least change with a quotient of 0.82. In the UVB irradiated lenses with a quotient value of 0.78, the least cataractogenic effect was detected, followed by the quotient of the UVA irradiated lenses with 0.78 as a quotient. The 460 nm-irradiated lenses with a quotient of 0.69 reveal the greatest cataractogenic effect. The error bars indicate the standard error of the mean.

The smaller the quotient Q_DFI,_ the brighter the dark field images. A bright dark field image indicates a structural change of the lens and, therefore, cataract development. In Figure 6 the calculated quotient Q_DFI_ is presented for each radiation wavelength and the control group with a given standard error of the mean. The closer the quotient is to one, the less cataractogenic development happened to the lens due to irradiation. The closer the quotient is to zero, the more cataract formation occurred. Lenses irradiated with 460 nm have the smallest quotient value of 0.69 and, therefore, the most cataract development. UVA irradiated lenses have the second-largest change in dark field images before and after irradiation with a quotient of 0.74, followed by the UVB irradiated lenses with a quotient of 0.78. The least amount of change had the control group with a quotient of 0.82. This change in the control group represents the aging effect of the lenses, which is also mentioned in the transmission measurements.

## 4. Discussion

Generally, radiation of all three wavelengths (311 nm, 370 nm, and 460 nm) reveals an effect on the porcine eye lenses. In these experiments, the strongest effect on the lenses is observed with 460 nm irradiation and is, therefore, most cataractogenic. According to these measurements, UVB and UVA radiation is less cataractogenic than 460 nm light. Both techniques to examine cataracts, the transmission measurements, and dark field analysis, indicate that irradiation with 460 nm has the greatest effect on porcine lenses regarding cataract development. By comparing the transmission measurements and the dark field method, differences in the measurement data become identifiable. The transmission measurement indicates that UVB radiation is more cataractogenic than UVA radiation, whereas the dark field method ranks UVA radiation as more cataractogenic than UVB radiation. The published literature suggests otherwise. Literature data indicate UVB radiation has the greatest impact on cataract development, which strongly declines by increased wavelength [18,42]. The kind of transmission decline due to cataract development detected in this experiment seems to comply with the literature. In both, the effect of transparency loss is most significant at the blue spectrum range and less in the red spectrum (see Figure 3 and Figure 4). This is also presented in Dillon et al. (1999) [43].

The transparency measurements result from a single dot measurement from the center of the lens. Other measurement patterns have not been realized because of the different refractive properties of the lens. Hence, this single transparency measurement per lens might not represent the overall condition of the lens. With $\sum_{i=1}^{n}p_{i}\left(\mathrm{before}\right)$ and $\sum_{i=1}^{n}p_{i}\left(\mathrm{after}\right)$, the whole lens condition is taken into account by the dark field image analysis, which describes the cataract development more precisely. However, the segmentation of the lens is improvable. Here, a threshold is applied to segment the lens from the background. More complex methods like deep learning could improve this image analysis process with less error detecting the lens.

In this experiment, the main factor is the cataract development due to different radiations. Nevertheless, considering the different effects of radiation compared to the literature, other effects must be considered. One factor might be bacterial growth during the 24 h experiment duration despite the usage of GVPC and intense irradiation, which should mitigate bacterial growth [44]. Another impact might be the overall experiment time because lens tissue degenerates after 5 h after lens extraction [45].

The methods employed to measure transparency, more specifically cataract development, are limited to greater structural cataract formations. However, cataract formation starts earlier on a molecular basis, which is detectable by fluorescence methods. Hence, the detection with these methods of greater magnitude is relatively late by considering there is no treatment option other than operational lens removal and artificial replacement.

A well-known fact is that UV irradiation generates reactive oxygen species, which leads to cell damage or even apoptosis followed by cataract formation [46,47,48]. There is no previous report on cataract formation with blue light and its mechanism. Some experiments on different human cells suggest that blue light causes oxidative stress because of endogen photosensitizers [49,50,51,52]. This mechanism could be a possible pathway to how blue light affects the lens.

The porcine eye is anatomically very close to the human eye. The retina vascularization is similar, and the lens thickness, shape, and size are comparable [39,53]. Moreover, the transmission of the porcine lens is also close to the human lens [38]. These similarities make the porcine eye currently suitable as an experimental model for the human eye.

To get more data and statements regarding cataract development, which focus on constant exposure to “the new and hidden blue light sources”, additional research and further experiments are necessary. The use of animal lenses is sufficient for the first proof of concept. However, it is undeniable that human lenses are still different and might react differently than animal lenses despite the similarities. Our work is limited to ex-vivo porcine lenses. We were not able to perform in-vivo measurements in our laboratory. This work shall inspire researchers with a focus on health and the environment, in general, to investigate cataract development, especially regarding the increasing exposure to blue light.

## 5. Conclusions

In this study, blue light has a measurable effect on cataract development. Transparency measurements and dark field image analysis rank blue light as more cataractogenic than UVB and UVA radiation. The literature nowadays is in contrast to this finding.

## Data Availability

The data presented in this study are available on request from the corresponding author.
